# Supplementary material for: Glucagon receptor signaling is indispensable for the healthspan effects of caloric restriction in aging male mice
Source: GeroScience. 2025 Sep 25;48(1):31–53. doi: 10.1007/s11357-025-01899-w (PMC12972411; doi:10.1007/s11357-025-01899-w)
Supplement: Supplementary file 1 — (DOCX 51.8 KB) [file 11357_2025_1899_MOESM1_ESM.docx]

**Supplemental Data Figure Legends:**

**Figure S1: Food Intake.** Ad libitum 24-hour food intake in WT and Gcgr KO male mice at 4- (A), 6- (B), and 12- (C) months of age. Gcgr KO: global glucagon receptor knockout, WT: wildtype littermate controls. Independent t-test. NS, not significant, data are means ± SEM.

**Figure S2: Whole Body Energy Expenditure and Substrate Utilization.** Energy expenditure (EE, kcal/ hour/ kg lean mass) over 24 hours, light and dark cycle average energy expenditure in 6-month-old (A-C: n= 5-7 mice per group) and 12-month-old (D-F: n= 5-9 mice per group) male mice. Corresponding respiratory quotient (RQ) in 6 month (G-I) and 12 month (J-L) old mice. Gcgr KO: global glucagon receptor knockout, WT: wildtype littermate controls. AL, ad libitum fed; CR, calorie restricted (15% initiated at 4.5 months of age). ^a,b^Superscript letters that differ indicate differences, P≤ 0.01; two-way ANOVA with Tukey’s adjustment for multiple comparisons, data are means ± SEM.

**Figure S3: Insulin Tolerance.** Insulin sensitivity, as assessed by an insulin tolerance test (0.5 IU/kg, intraperitoneal) at 6- (A-D: n= 12-19 male mice per group) and 17- (E-F: n= 5-11 male mice per group) months of age. Ad libitum (AL), 15% calorie restricted (15% CR). ^a,b^Superscript letters that differ indicate differences, P≤ 0.01; two-way ANOVA with Tukey’s adjustment for multiple comparisons. NS, not significant, data are means ± SEM.

**Figure S4: Liver Glycogen and cyclic AMP content.** Liver glycogen (mg/g liver) in 6- (A, n=12-19) and 17- (B, n= 6-11) month old male mice and liver cyclic AMP (cAMP) in 6- (C, n=12-19) and 17- (D, 6-10) month old male mice. AL, ad libitum fed; CR, calorie restricted (15% initiated at 4.5 months of age). Gcgr KO: global glucagon receptor knockout, WT: wildtype littermate controls. ^a,b^Superscript letters that differ indicate differences, P< 0.05; two-way ANOVA with Tukey’s adjustment for multiple comparisons, data are means ± SEM.

**Figure S5: Glucagon receptor agonism only increases liver cAMP acutely**

Liver cyclic AMP (cAMP) content in wildtype male C57BL/6J mice treated with a long-acting glucagon analogue A) Acutely (Single does GCGA, 1.5 nmol/kg BW subcutaneous) and B-C) Long-term (GCGA, 3 nmol/kg BW subcutaneous, three times per week for 4 weeks). Tissues were collected from 6-month-old (A-B) and 17-month-old (C) male mice. n= 5-7 mice per group; unpaired t-test, data are means ± SEM.

**Figure S6: Hepatic Gene Expression.**

Liver mRNA expression of glucagon receptor (Gcgr, A&E), phosphoenolpyruvate carboxykinase (Pck1, B&F), glucose 6 phosphatase (Gcpc1, C&G), mechanistic target of rapamycin (Mtor, D&H), acetyl-Coenzyme A carboxylase alpha (Acaca, I&M), carnitine palmitoyl transferase 1a (Cpt1a, J&N), peroxisome proliferator activated receptor alpha (Ppara, K&O), and nuclear receptor subfamily 1, group D, member 1 (Nr1d1, L&P) in 6- (n= 11-19) and 17- (n= 6-10) month old male mice. ^a,b^Superscript letters that differ indicate differences, P≤ 0.01; two-way ANOVA with Tukey’s adjustment for multiple comparisons. NS, not significant, data are means ± SEM.

**Figure S7: Physical Function.** Physical function as assessed by time to fall in seconds on a Rotarod performance test in 6- (A, n= 9-15) and 17- (B, n=6-11) month old male mice. AL, ad libitum fed; CR, calorie restricted (15% initiated at 4.5 months of age). Gcgr KO: global glucagon receptor knockout, WT: wildtype littermate controls. ^a,b^Superscript letters that differ indicate differences, P< 0.05; two-way ANOVA with Tukey’s adjustment for multiple comparisons, data are means ± SEM.

**Figure S8:** A) Full Western Blot Membranes from Figures 5C-D. Phosphorylated and total Ribosomal S6 Protein blots. B) Full Western Blot Membranes from Figures 5A-B. Phosphorylated and total AMPK blots.

**Figure S9:** Full Western Blot Membranes from Figure 6, Phosphorylated and total Ribosomal S6 Protein blots.

| **Supplemental Data Table 1.** Pathology at Time of Death in Gcgr KO mice | Gcgr KO (n=9) | % Incidence |
| --- | --- | --- |
| **Pancreas** |  |  |
| Islet cell carcinoma | 5 | 55% |
| **Liver** |  |  |
| Hepatic Metastasis of Islet cell carcinoma | 1 | 11% |
| **Kidney** |  |  |
| Moderate glomerular amyloidosis | 1 | 11% |
| Mild to moderate post-mortem autolysis | 3 | 33% |
| **Heart** |  |  |
| None |  | 0% |
| **Lung** |  |  |
| Mild Focal alveolar histiocytosis | 1 | 11% |
| **Stomach** |  |  |
| Wall Discoloration | 1 | 11% |
| Distension | 2 | 22% |
| **Cecum** |  |  |
| Enlarged | 1 | 11% |
| **Gallbladder** |  |  |
| None |  | 0% |
| **Spleen** |  |  |
| Extramedullary hematopoiesis | 1 | 11% |
| **Prolapse (of any organ)** |  |  |
| Rectal | 1 | 11% |
| Penile | 1 | 11% |
| **Abdomen** |  |  |
| Cranioventral Round cell neoplasia | 1 | 11% |
| Distension | 1 | 11% |
| **No significant lesions detected (including the middle ear)** | 3 | 33% |

| **Phenotype Category** | **Response to CR** | | | |  | **Response to CR** | |  | **Effect of Glucagon Receptor Agonist** | |
| --- | --- | --- | --- | --- | --- | --- | --- | --- | --- | --- |
|  | Littermate WT | | Gcgr KO | |  | Littermate WT | Gcgr Liver KO |  | Wildtype C57Bl/6J | |
|  | Young | Aged | Young | Aged |  | Aged | Aged |  | Young | Aged |
| Glucose Homeostasis | ↑ | ↑ | ↔ | ↔ |  |  | |  |  | |
| Lipid Homeostasis | ↑ | ↑ | ↔ | ↔ |  |  |  |  |  |  |
| Physical Function | ↑ | ↑ | ↔ | ↔ |  |  |  |  |  |  |
| Body Weight | ↓ | ↓ | ↓ | ↔ |  |  |  |  |  |  |
| Fat Mass | ↓ | ↓ | ↔ | ↔ |  |  |  |  |  |  |
| Whole Body Energy Expenditure  (Light Cycle) | ↔ | ↔ | ↔ | ↓ |  |  |  |  |  |  |
| Whole Body Energy Expenditure  (Dark Cycle) | ↓ | ↔ | ↔ | ↔ |  |  |  |  |  |  |
| Respiratory Quotient (Light Cycle) | ↓ | ↓ | ↓ | ↓ |  |  |  |  |  |  |
| Respiratory Quotient (Dark Cycle) | ↓ | ↔ | ↔ | ↔ |  |  |  |  |  |  |
| mTOR activity |  | | | |  | ↓ | ↔ |  | ↓ | ↓ |
| AMPK Activation |  |  |  |  |  | ↔ | ↓ vs WT Regardless of diet |  | ↔ | ↔ |

**Supplemental Table 2. Comparison of key phenotypes in response to CR and pharmacologic glucagon receptor agonism**

**Supplemental Table 3. 2-Way ANOVA results for genotype, diet, and their interaction for all significant outcomes.**

| **Data Figure** | **2-Way ANOVA P Value** | | |
| --- | --- | --- | --- |
|  | Genotype | Diet | Genotype x Diet Interaction |
| Figure 2B. Body Weight 6 Months | P=0.9549 | P<0.001 | P=0.2081 |
| Figure 2C. Fat Mass 6 Months | P<0.0001 | P<0.0001 | P=0.0344 |
| Figure 2D. Body Weight 12 Months | P=0.0083 | P<0.0001 | P=0.0014 |
| Figure 2E. Fat Mass 12 Months | P<0.0001 | P=0.0001 | P=0.0027 |
| Figure 2F. Body Weight 17 Months | P=0.0527 | P=0.0002 | P=0.0152 |
| Figure 2G. Fat Mass 17 Months | P=0.0003 | P=0.0056 | P=0.0106 |
| Figure 3A. Basal Glucose 6 Months | P<0.0001 | P<0.0001 | P=0.0377 |
| Figure 3B. Basal Insulin 6 Months | P=0.0019 | P=0.1062 | P=0.2197 |
| Figure 3D. OGTT AUC 6 Months | P<0.0001 | P<0.0001 | P=0.0002 |
| Figure 3E. Basal Glucose 17 Months | P<0.0001 | P=0.0017 | P=0.0024 |
| Figure 3F. Basal Insulin 17 Months | P=0.0051 | P=0.0046 | P=0.0003 |
| Figure 3H. OGTT AUC 17 Months | P<0.0001 | P=0.0095 | P=0.0032 |
| Figure 3I HOMA-IR 6 Months | P=0.0003 | P=0.0092 | P=0.0583 |
| Figure 3J. Oral Glucose Stimulated Insulin 6 Months | P=0.5911 | P=0.0187 | P=0.1693 |
| Figure 3L. Delta OGTT AUC 6 Months | P<0.0001 | P<0.0001 | P=0.2490 |
| Figure 3M HOMA-IR 17 Months | P=0.0007 | P=0.0009 | P=0.0001 |
| Figure 3N. Oral Glucose Stimulated Insulin 17 Months | P=0.0403 | P=0.0015 | P=0.0043 |
| Figure 3P. Delta OGTT AUC 17 Months | P=0.2439 | P=0.2041 | P=0.4617 |
| Figure 4A. Liver Triglyceride 6 Months | P=0.4837 | P=0.0007 | P=0.0167 |
| Figure 4B. Serum Triglyceride 6 Months | P=0.0542 | P=0.0004 | P=0.0193 |
| Figure 4C. Serum Cholesterol 6 Months | P<0.0001 | P=0.5182 | P=0.0009 |
| Figure 4D. Liver Triglyceride 17 Months | P=0.0117 | P=0.0077 | P=0.0147 |
| Figure 4E. Serum Triglyceride 17 Months | P=0.1264 | P=0.2725 | P=0.0084 |
| Figure 4F. Serum Cholesterol 17 Months | P=0.5944 | P=0.0897 | P=0.0849 |
| Figure 5A. mTOR Activation WT vs Gcgr ^Hep-/-^ | P=0.0039 | P=0.0388 | P=0.3904 |
| Figure 5C. AMPK Activation WT vs Gcgr ^Hep-/-^ | P=0.0007 | P=0.2200 | P=0.829 |
| Supplemental Figure 2B. Light Cycle EE 6 Months | P=0.0117 | P=0.0038 | P=0.6609 |
| Supplemental Figure 2C. Dark Cycle EE 6 Months | P=0.0150 | P=0.0005 | P=0.4723 |
| Supplemental Figure 2E. Light Cycle EE 12 Months | P=0.6421 | P=0.0003 | P=0.4486 |
| Supplemental Figure 2H. Light Cycle RQ 6 Months | P=0.3778 | P<0.0001 | P=0.2859 |
| Supplemental Figure 2I. Dark Cycle RQ 6 Months | P=0.9380 | P=0.0004 | P=0.6711 |
| Supplemental Figure 2K. Light Cycle RQ 12 Months | P=0.0204 | P<0.0001 | P=0.1770 |
| Supplemental Figure 3B. ITT AUC 6 months | P<0.0001 | P<0.0001 | P=0.0119 |
| Supplemental Figure 3F. ITT AUC 17 months | P=0.0089 | P=0.0263 | P=0.0446 |
| Supplemental Figure 4A. Liver Glycogen 6 Months | P=0.2257 | P=0.0029 | P=0.0162 |
| Supplemental Figure 6A. Gcgr mRNA 6 Months | P<0.0001 | P=0.0763 | P=0.2613 |
| Supplemental Figure 6B. Pck1 mRNA 6 Months | P<0.0001 | P=0.3188 | P=0.0274 |
| Supplemental Figure 6C. G6pc1 mRNA 6 Months | P=0.8792 | P<0.0001 | P=0.5275 |
| Supplemental Figure 6E. Gcgr mRNA 17 Months | P<0.0001 | P=0.4529 | P=0.2274 |
| Supplemental Figure 6H. Mtor mRNA 17 Months | P=0.0756 | P=0.0061 | P=0.9372 |
| Supplemental Figure 6I. Acaca mRNA 6 Months | P=0.0025 | P=0.0353 | P=0.7150 |
| Supplemental Figure 6J. Cpt1a mRNA 6 Months | P=0.0415 | P=0.1236 | P=0.1698 |
| Supplemental Figure 6K. Ppara 6 Months | P=0.1217 | P=0.0403 | P=0.4128 |
| Supplemental Figure 6L. Nr1d1 mRNA 6 Months | P=0.5241 | P=0.0017 | P=0.6526 |
| Supplemental Figure 6M. Acaca mRNA 17 Months | P=0.0119 | P=0.1837 | P=0.2843 |
| Supplemental Figure 6P. Nr1d1 mRNA 17 Months | P=0.5798 | P=0.5067 | P=0.0027 |
| Supplemental Figure 7A. Rotarod time to fall 6 Months | P=0.1607 | P=0.1273 | P=0.0290 |
| Supplemental Figure 7B. Rotarod time to fall 17 Months | P=0.5568 | P=0.0135 | P=0.0474 |
