# Supplementary material for: Glucagon receptor signaling is indispensable for the healthspan effects of caloric restriction in aging male mice
Source: GeroScience. 2025 Sep 25;48(1):31–53. doi: 10.1007/s11357-025-01899-w (PMC12972411; doi:10.1007/s11357-025-01899-w)

Supplemental Figure 1

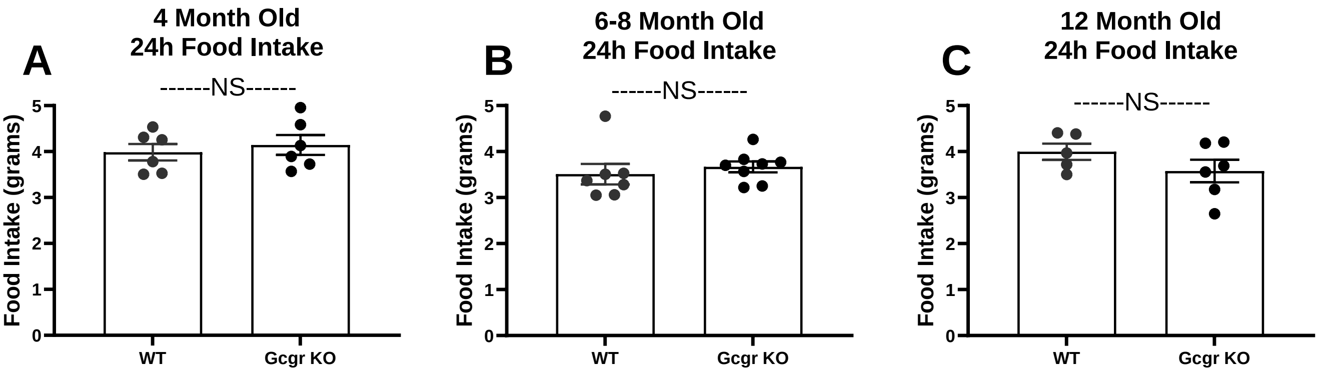

Supplemental Figure 2

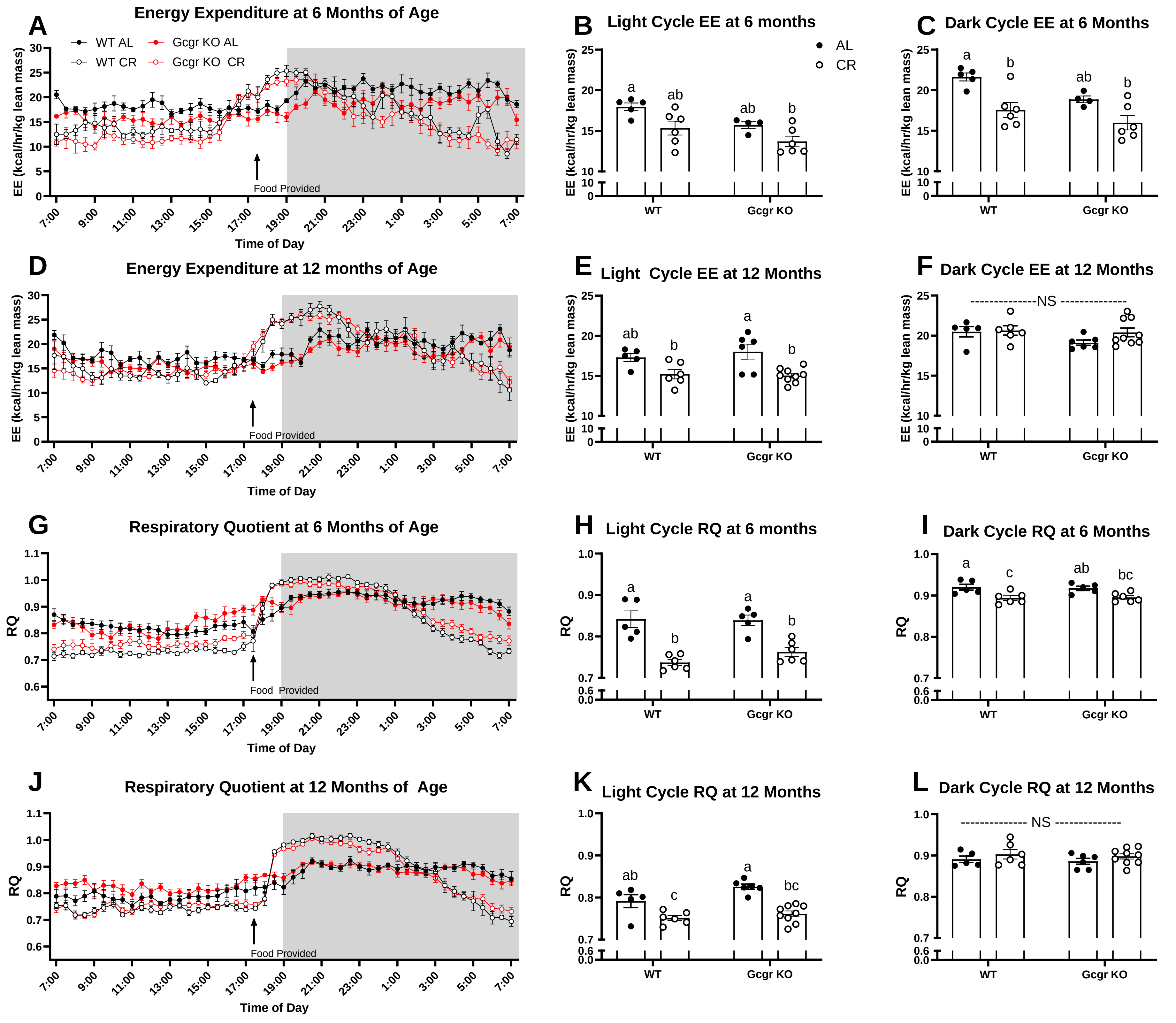

Supplemental Figure 3

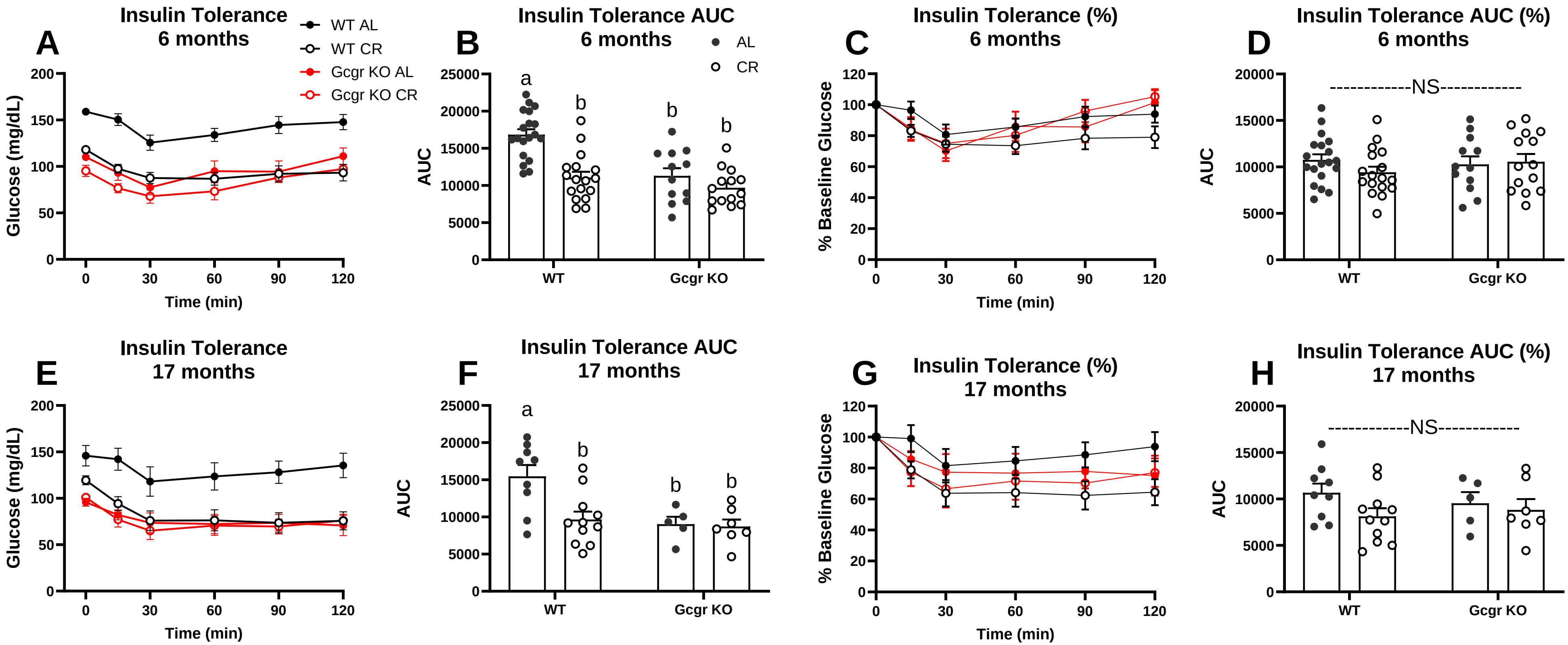

Supplemental Figure 4

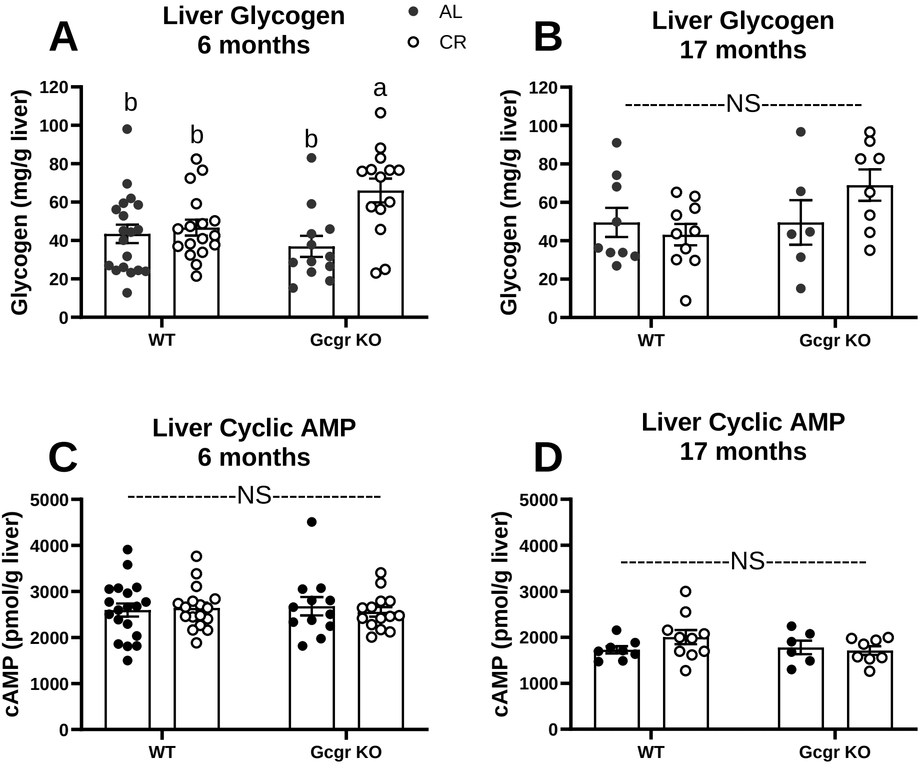

Supplemental Figure 5

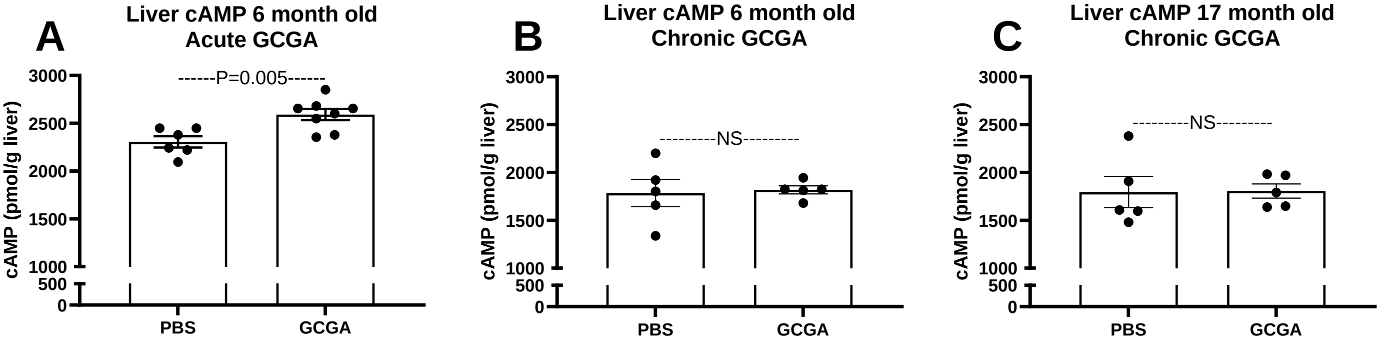

Supplemental Figure 6

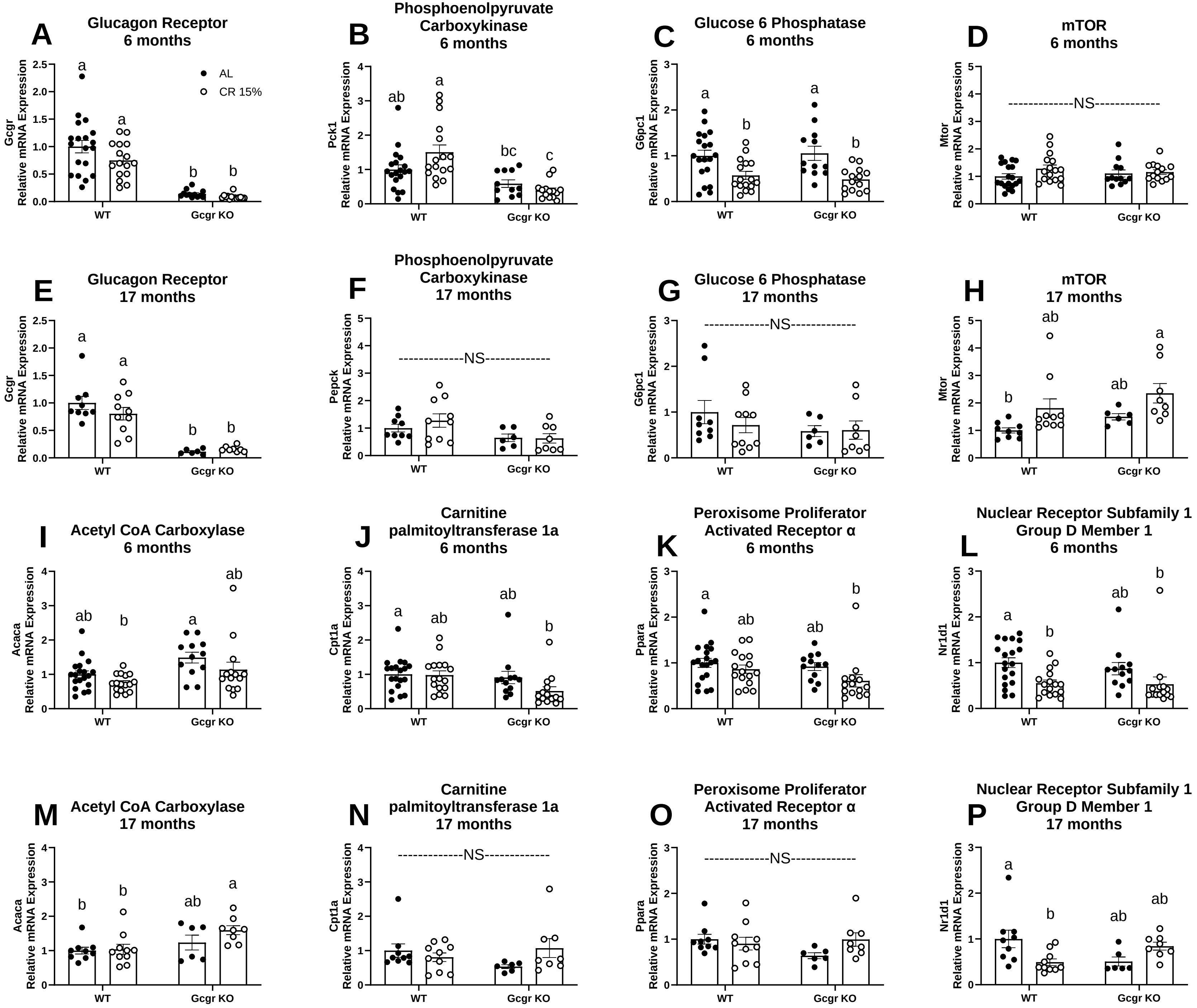

Supplemental Figure 7

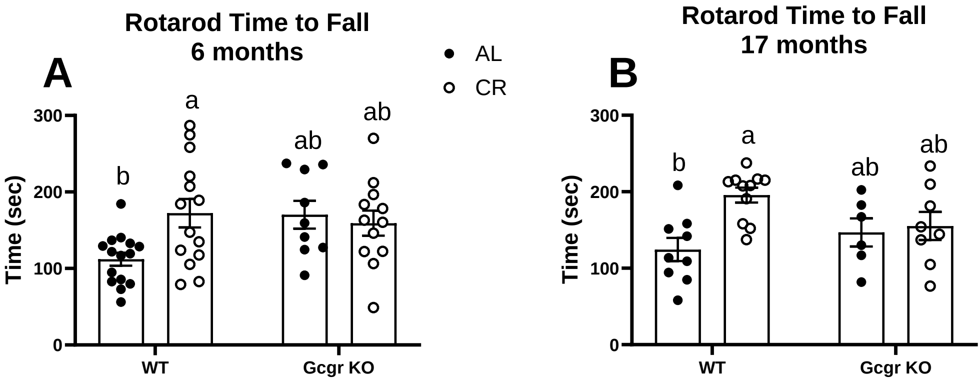

Supplemental Figure 8A: Full membranes from Figure 5A-B: Phospho and Total S6

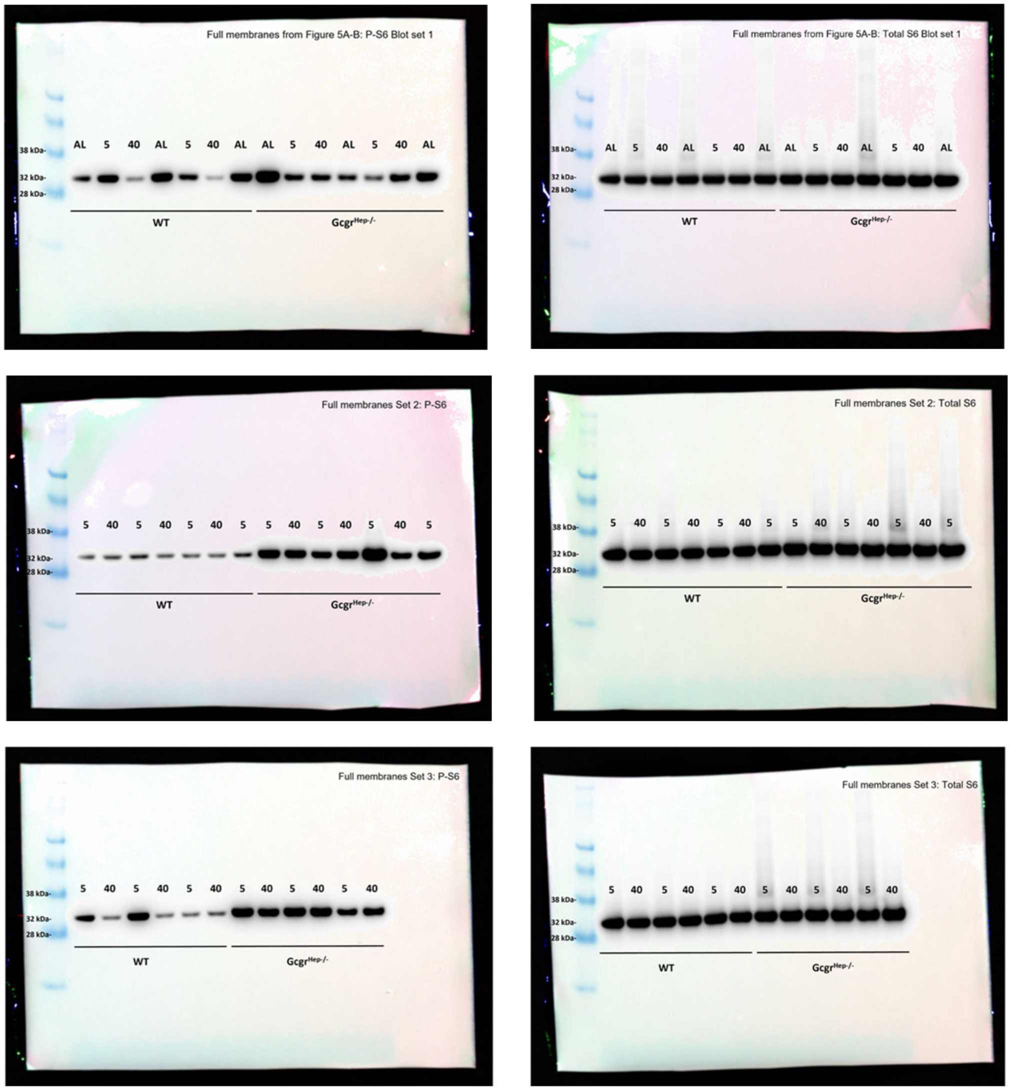

Supplemental Figure 8B: Full membranes from Figure 5C-D: Phospho and Total AMPK

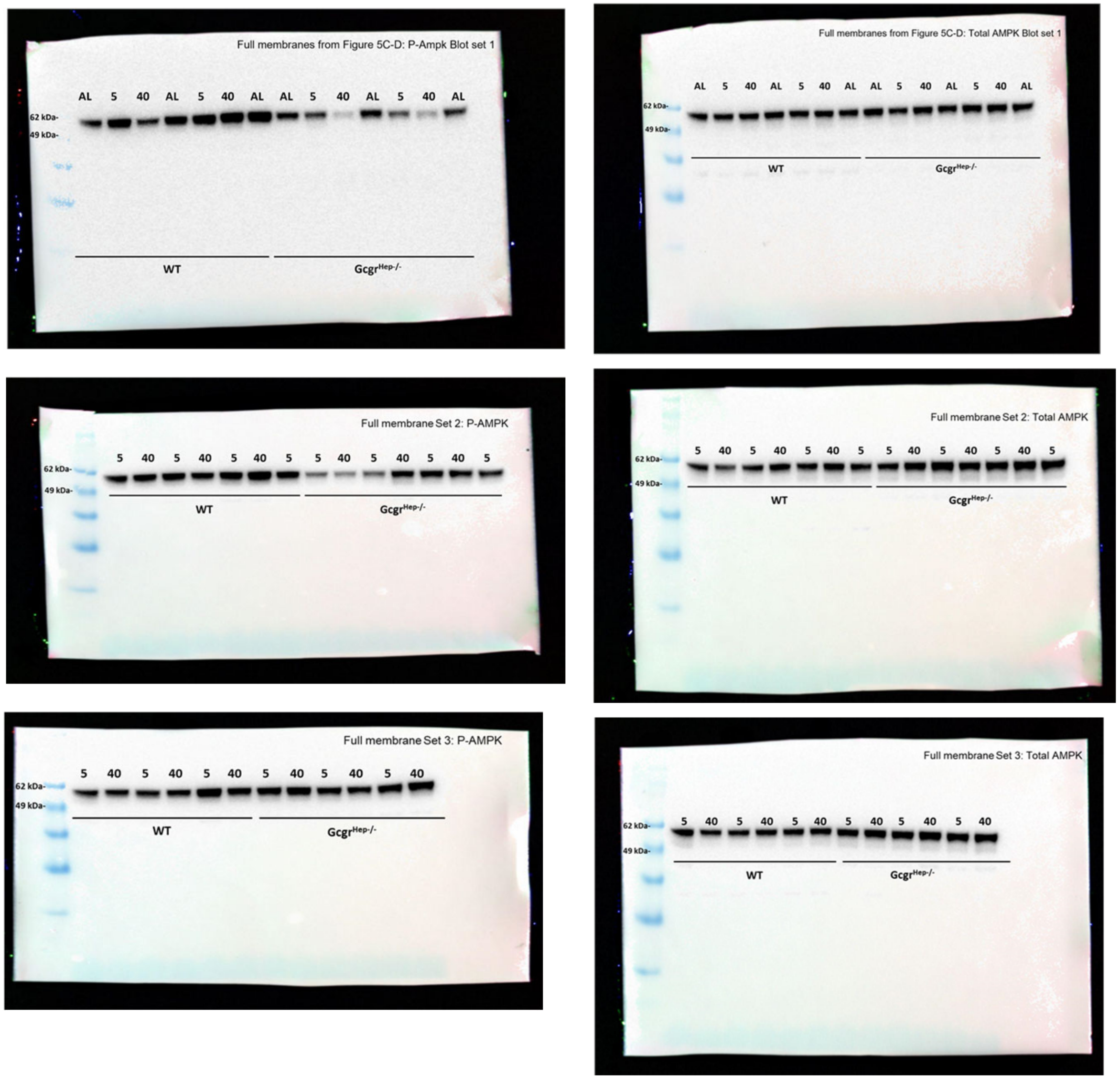

Supplemental Figure 9

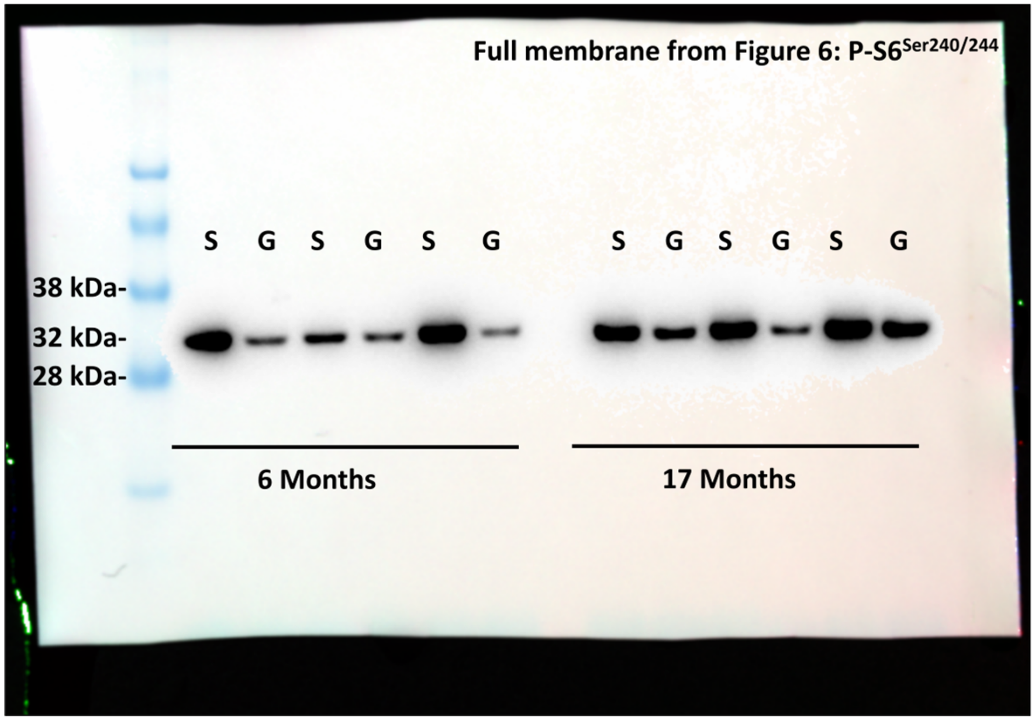

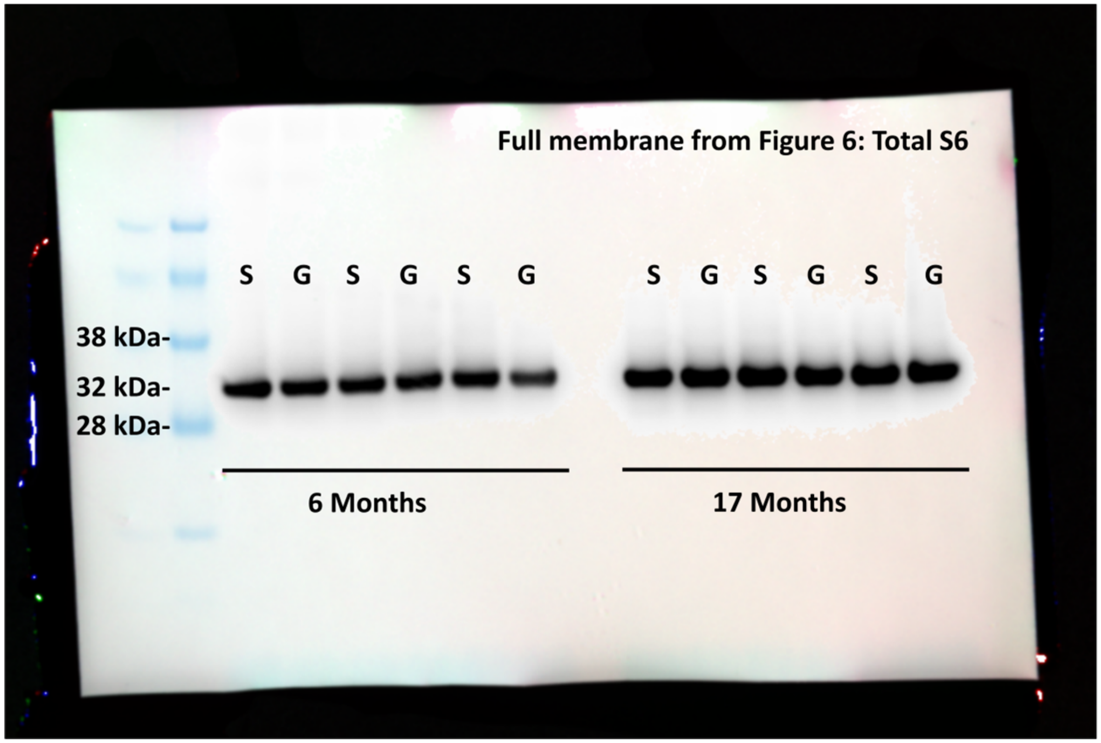

Supplement: Supplementary file 2 — (PDF 3.18 MB) [file 11357_2025_1899_MOESM2_ESM.pdf]
